# Interleukin-38: A Candidate Biomarker for Disease Severity in Advanced Steatotic Liver Disease

**DOI:** 10.3390/cells15030280

**Published:** 2026-02-02

**Authors:** Valeria Wagner, Michael Mederer, Barbara Enrich, Veronika Cibulkova, Johanna Piater, Andreas Zollner, Rebecca Giquel-Fernandes, Herbert Tilg, Maria Effenberger

**Affiliations:** Department of Internal Medicine I, Gastroenterology, Hepatology, Endocrinology & Metabolism, Medical University of Innsbruck, Christoph-Probst-Platz 1, 6020 Innsbruck, Austria

**Keywords:** IL-38, IL-1 cytokine family, alcohol-associated liver disease (ALD), metabolic dysfunction-associated alcohol-related liver disease (metALD), hepatic decompensation, biomarker

## Abstract

Background: Interleukin-38 (IL-38) is an anti-inflammatory IL-1—family cytokine implicated in limiting tissue injury by its anti-inflammatory character. We evaluated the diagnostic discrimination and prognostic relevance in steatotic liver disease (SLD). Methods: We conducted a prospective, monocentric cohort analysis of 184 patients with SLD (*n* = 176) and healthy controls (*n* = 8). We tested group differences using Mann–Whitney U or Kruskal–Wallis; determined diagnostic quality using ROC curves. Logistic regression was used to assess the relationship with decompensation. Associations with MELD and routine laboratory parameters were modeled using Spearman correlation and linear regression. We analyzed survival using Kaplan–Meier and Cox regression. Findings: IL-38 concentrations were found to be higher in decompensated (*n* = 94) than in compensated patients (*n* = 82) (*p* < 0.001). MELD was positively associated with IL-38 (*p* < 0.001; 95% CI 0.057–0.120). This corresponds to a 9.2% increase in IL-38 per 1-point increase in MELD (95% CI 5.9–12.7%). IL-38 correlated positively with the MELD score (*p* < 0.001) and with bilirubin/AST/LDH. In the combination model (MELD + IL-38 ± CRP), a very good AUC ≈ 0.92 was achieved. Conclusion: IL-38 reflects the severity of steatotic liver disease and is therefore a potentially predictive biomarker for early risk stratification and therapy monitoring.

## 1. Introduction

Liver diseases, including alcohol-associated liver disease (ALD), metabolic dysfunction–associated steatotic liver disease (MASLD), and the combined phenotype of metabolic dysfunction–associated and alcohol-associated liver disease (metALD), represent among the leading causes of chronic morbidity worldwide and constitute a major and growing public health burden [1,2]. Despite their distinct etiological triggers, these disease entities share core pathophysiological mechanisms that drive the progressive transition from simple steatosis to steatohepatitis, fibrosis, and ultimately cirrhosis with its associated complications. Chronic inflammation arises from sustained activation of innate immune pathways, including macrophage-driven cytokine signaling, oxidative stress responses, and inflammasome-associated cascades, which together perpetuate hepatocellular injury, dysregulated wound healing, and fibrogenic repair programs [3]. In ALD, this inflammatory milieu is primarily initiated by alcohol-induced cellular stress and disruption of the gut–liver axis with increased translocation of bacterial products and endotoxin exposure [4,5], whereas in MASLD, visceral adipose tissue dysfunction, insulin resistance, lipotoxicity, and chronic low-grade systemic inflammation critically amplify hepatic inflammatory responses and metabolic injury [6,7].

Within this complex inflammatory network, the interleukin-1 (IL-1) cytokine family has emerged as a central regulatory axis in innate immune activation, tissue injury, and fibrogenesis [8,9]. Interleukin-38 (IL-38) represents one of the most recently identified members of the IL-1 cytokine family and is characterized by predominantly anti-inflammatory and immunomodulatory properties [10]. By modulating the activity of T lymphocytes and myeloid cell populations, IL-38 attenuates Th17-driven inflammatory responses and limits excessive immune activation. In addition, tolerogenic effects have been described through the regulation of dendritic cell maturation and regulatory T cell (Treg) differentiation, as well as through the inhibition of pro-inflammatory macrophage polarization and cytokine production [11,12]. Furthermore, IL-38 exerts cytoprotective and anti-inflammatory effects in epithelial and mesenchymal cells by preserving tissue integrity, limiting cellular stress responses, and promoting reparative and regenerative processes [13]. Protective roles for IL-38 have been reported in several autoimmune conditions as well as in infectious, cardiovascular, and central nervous system diseases, highlighting its broad immunoregulatory potential across organ systems [14]. Nevertheless, critical aspects of IL-38 receptor interactions, downstream signaling mechanisms, and context-dependent biological effects remain incompletely defined, and the precise role of IL-38 in chronic inflammatory liver disease is still poorly understood [15].

Initial clinical observations indicate that circulating IL-38 concentrations are elevated in MASLD and are associated with insulin resistance, hepatic transaminase activity, and dyslipidemia, suggesting a potential link between IL-38 and metabolic liver injury [16]. Preclinical studies in experimental models of obesity and hepatic steatosis further demonstrate attenuation of inflammatory signaling and insulin resistance, together with a reduction in hepatic lipid accumulation, in part mediated by AMP-activated protein kinase–dependent activation of autophagy, modulation of lipid metabolism, and mitigation of endoplasmic reticulum stress [17]. These experimental findings support a mechanistic role for IL-38 in metabolic homeostasis and hepatocellular stress responses and provide a biological rationale for its investigation in human steatotic liver disease.

Collectively, the available evidence suggests that IL-38 represents a promising biomarker and a potentially therapeutically relevant target in chronic inflammatory and metabolic liver disease. However, human data remain scarce, particularly in the setting of inflammatory metabolic liver pathology and advanced steatotic liver disease [18]. In particular, the relationship between circulating IL-38 concentrations, disease severity, hepatic decompensation, and clinical outcomes has not been systematically examined. The objective of the present study is therefore to characterize circulating IL-38 concentrations in a well-defined cohort with steatotic liver disease (SLD) and to investigate their diagnostic and prognostic relevance in order to evaluate the potential clinical utility of IL-38 as a biomarker and adjunctive risk stratification parameter in this growing patient population.

## 2. Materials and Methods

### 2.1. Study Design

This study prospectively analyzed IL-38 in 176 patients with alcohol-associated liver disease (ALD), metabolic dysfunction and alcohol associated liver disease (metALD), metabolic dysfunction-associated steatosis liver disease (MASLD) regularly seen in the hepatology outpatient clinic of the Medical University of Innsbruck.

In addition, a healthy control cohort consisting of 8 individuals was established. IL-38 concentrations were determined once at the time of the clinical consultation, either during an inpatient admission or in the context of a scheduled outpatient visit. Routine laboratory analyses were performed concurrently with IL-38 sampling. Clinical data were collected at the same time point and included, among other variables, the current clinical condition and the presence of hepatic decompensation at the time of blood collection. Patients were recruited between 2018 and 2020 at the Department of Internal Medicine I, Gastroenterology, Hepatology, Endocrinology and Metabolism, Medical University of Innsbruck.

The hospital’s medical patient administration system was used for the systematic collection and documentation of serum IL-38 concentrations (ng/mL), demographic characteristics (including sex, body mass index, and related variables), disease classification, indicators of disease severity (including decompensation status and Model for End-Stage Liver Disease [MELD] score), as well as routine laboratory parameters.

### 2.2. IL-38 Quantification

IL-38 concentrations were quantified using a commercially available sandwich enzyme-linked immunosorbent assay (ELISA) (Human IL-38/IL-1F10 DuoSet ELISA, DY9110-05; R&D Systems, Minneapolis, MN, USA) according to the manufacturer’s instructions. Briefly, 96-well microplates were coated overnight at room temperature with capture antibody specific for human IL-38. After blocking to prevent nonspecific binding, serum or plasma samples and recombinant IL-38 standards were added in duplicate and incubated to allow antigen binding. Plates were then washed and incubated with a biotinylated detection antibody, followed by streptavidin–horseradish peroxidase conjugate. Signal development was achieved using the tetramethylbenzidine substrate and stopped with an acidic stop solution. Optical density was measured at 450 nm with wavelength correction at 570 nm. IL-38 concentrations were calculated from a standard curve generated by four-parameter logistic regression. Samples with concentrations outside the assay range were appropriately diluted and reanalyzed.

### 2.3. Clinical Definitions

In the present study, hepatic decompensation was defined as a clinically documented transition from the compensated to the decompensated stage of liver disease, in accordance with the currently valid Baveno VII consensus criteria. Accordingly, patients were classified as decompensated if, at the time of IL-38 sampling, they exhibited at least one of the following clinical manifestations: ascites, variceal bleeding, clinically overt hepatic encephalopathy, or jaundice, reflecting clinically relevant deterioration of hepatic function [19]. All patients categorized as decompensated were experiencing an active episode of decompensation at the time of clinical assessment.

Clinically manifest hepatic encephalopathy (HE) was defined as “overt hepatic encephalopathy” and operationalized according to the West Haven classification. Episodes of encephalopathy were classified as overt when they reached West Haven grade II or higher, that is, conditions characterized by clearly recognizable neuropsychiatric abnormalities, including disorientation, behavioral changes, asterixis, and progressive impairment of alertness up to stupor or coma in severe cases. The diagnosis was established primarily on clinical grounds based on detailed medical documentation, with systematic consideration of competing causes of acute encephalopathy and potential precipitating factors, in particular infections, gastrointestinal bleeding, constipation, electrolyte disturbances, deterioration of renal function, and the use of sedative medications [19].

ALD was defined by the presence of chronic liver disease with relevant alcohol consumption (>50 g/day in women and >60 g/day in men) as the primary etiological factor, in the absence of a dominant metabolic cause of hepatic steatosis. Relevant alcohol consumption was defined according to established international consensus criteria. Patients with alcoholic hepatitis were excluded from the study [20].

MetALD was defined in patients with hepatic steatosis and concurrent relevant alcohol consumption (>20 g/day in women and >30 g/day in men) in combination with the presence of metabolic dysfunction, such as overweight or obesity, type 2 diabetes mellitus, arterial hypertension, or dyslipidemia. Classification was performed in accordance with the current nomenclature, distinguishing MASLD from ALD [21]. Patients with alcoholic hepatitis, defined according to the current criteria as a distinct clinical syndrome characterized by newly developed jaundice in the context of ongoing harmful alcohol consumption and frequently accompanied by signs of hepatic decompensation, were not included in this analysis [22].

The diagnosis of MASLD was established according to the current international consensus definition. MASLD was defined by the presence of hepatic steatosis, detected either histologically, by imaging techniques, or by validated noninvasive markers, in combination with at least one cardiometabolic risk factor, including overweight or obesity, type 2 diabetes mellitus, arterial hypertension, or dyslipidemia. The diagnosis required the absence of relevant alcohol consumption and the exclusion of other competing causes of chronic liver disease. Patients with metabolic dysfunction and concomitant relevant alcohol consumption were classified as having metALD in accordance with the current nomenclature [23].

### 2.4. Statistical Analysis

All tests were two-tailed (α = 0.05) and confidence intervals (CI) are presented on a 95% level. Statistical analyses were performed using IBM SPSS Statistics version 31 (IBM Corporation, Armonk, NY, USA).

We tested differences in IL-38 levels using Mann–Whitney U (ALD/metALD vs. healthy individuals) and exploratively using Kruskal–Wallis (all four groups) with Bonferroni-corrected pair comparisons. We summarized the diagnostic specificity (ALD/metALD vs. healthy individuals) using ROC/AUC (nonparametric AUC, standard error, 95% CI); cut-offs were determined using the Youden index.

We compared IL-38 in compensated (*n* = 82) vs. decompensated (*n* = 94) patients using the Mann–Whitney U test. In addition, a binary logistic regression model was calculated with “logIL38” as the predictor. We assessed the discrimination of the models based on the AUC of the stored predicted probabilities; Youden-optimal and sensitivity-prioritized thresholds were taken from the ROC coordinates. We quantified the relationship between MELD and “logIL38” using Spearman correlations as well as linear regression. We calculated Spearman correlations between “logIL38” and standard laboratory tests (PT/INR, aPTT, albumin, total bilirubin, sodium, AST/ALT, GGT, ALP, LDH, CRP, creatinine, thrombocytes, leukocytes). We examined whether the IL-38 differs according to etiology and different decompensation stages in a logistic regression with “logIL-38”, group (ALD/metALD vs. MASLD), and their interaction term.

In ALD/metALD, we analyzed overall survival using Kaplan–Meier curves and Cox regressions using “logIL-38” as a continuous covariate.

To test for independence of global severity and/or inflammation, we calculated a multivariable logistic model with “logIL-38”, MELD, and ln(CRP). In addition, we determined partial Pearson correlations between ln(IL-38) and MELD, adjusting for ln(CRP) and etiology (ALD/metALD vs. MASLD).

For the survival analyses, we considered the subcohort with ALD/metALD. The MASLD cohort was not included in the survival analyses due to the low number of events. IL-38 was dichotomized for grouped comparisons either as median-based in “low” vs. “high” or based on the Youden cut-off from the decompensation ROC (0.138 ng/mL). Survival curves were plotted using Kaplan–Meier and compared using the log-rank test. In addition, the relationship between continuous logIL-38 and overall survival was examined in a Cox regression.

### 2.5. Ethical Considerations

The study was conducted in accordance with the Declaration of Helsinki and was approved by the local ethics Committee of the Medical University of Innsbruck (approval number: AN2017-0016 369/4.21). Written informed consent was obtained from all subjects.

## 3. Results

A total of *n* = 184 individuals were analyzed (ALD *n* = 59, metALD *n* = 39, MASLD *n* = 78, healthy controls *n* = 8); the proportion of decompensated individuals was 46.7% (86/184). IL-38 concentrations did not differ significantly between ALD, metALD, MASLD, and healthy individuals (Kruskal–Wallis *p* = 0.099) (Table 1). In contrast, IL-38 concentrations were higher in decompensated patients compared to compensated patients (median 0.221 vs. 0.135 ng/mL; Z = −4.761, *p* < 0.001; r = 0.33).

IL-38 correlated positively with laboratory parameters of advanced disease (including bilirubin ρ = 0.27; AST ρ = 0.16; LDH ρ = 0.18) and negatively with platelets (ρ = −0.31) and PT (ρ = −0.27) (Table 2).

In a linear regression model with ln(IL-38) as the dependent variable, the MELD score was significantly positively associated (B = 0.088; 95% CI 0.057–0.120; *p* < 0.001). This corresponds to a mean increase in IL-38 concentration of 9.2% per additional MELD point (95% CI 5.9–12.7%); an increase in MELD of 10 points was associated with approximately 2.4-fold higher IL-38 levels. A positive correlation between MELD and ln(IL-38) was consistently observed (ρ = 0.42; *p* < 0.001).

IL-38 was a significant predictor of decompensation in univariate logistic regression (OR 2.34, 95% CI 1.57–3.50; *p* < 0.001). In multivariate models, IL-38 lost its significance once MELD was included (MELD + logIL-38: MELD OR 1.92 [1.57–2.34], *p* < 0.001; logIL-38 OR 1.39 [0.83–2.32], *p* = 0.207; MELD + logIL-38 + lnCRP: MELD OR 1.90 [1.56–2.33], *p* < 0.001; logIL-38 OR 1.38 [0.82–2.32], *p* = 0.227; lnCRP OR 1.07 [0.97–1.17], *p* = 0.178); (Table 3).

**Figure 1:**
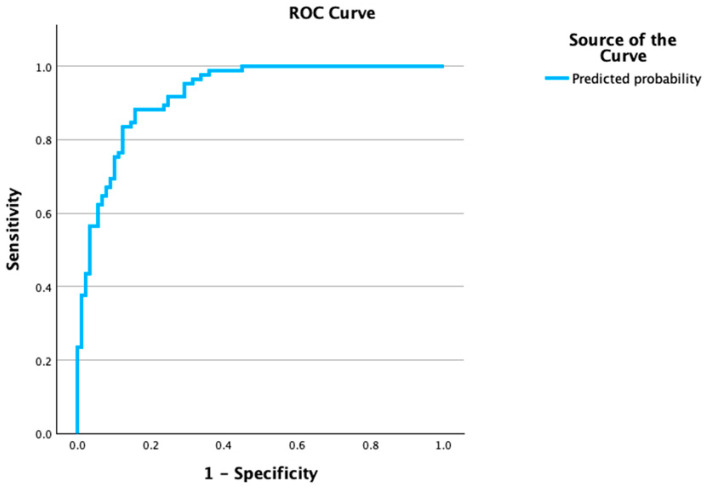
ROC curve for multivariable prediction of decompensation. Receiver operating characteristic (ROC) curve based on the logistic model, including MELD, log-transformed IL-38, and ln-CRP to classify decompensation (decompensated = 1). The model shows excellent discrimination with AUC = 0.925 (95% CI, 0.887–0.962; *p* < 0.001). The diagonal line indicates no discrimination (AUC = 0.5); sensitivity and specificity are plotted across all probability thresholds derived from the model’s predicted probabilities.

The ROC analysis for IL-38 to discriminate decompensation yielded an AUC of 0.713 (95% CI 0.525–0.901) with a Youden cut-off of ≈0.138 ng/mL (sensitivity 0.663, specificity 0.750). Multivariable models showed very good discrimination: MELD + logIL-38 with an AUC 0.920 (0.882–0.959) and Youden cut-off p* = 0.463 (sensitivity 0.882; specificity 0.854; estimated PPV = 0.84, NPV = 0.89 at prevalence 46.7%); MELD + logIL-38 + lnCRP with an AUC 0.925 (0.887–0.962) and p* = 0.435 (Sens 0.882; Spec 0.843; PPV ~0.83, NPV = 0.89), (Figure 1, Table 4).

Neither the median-based dichotomization of IL-38 nor the classification using the Youden cut-off showed a significant difference in the overall survival of ALD/metALD patients. Consistent with this, logIL-38 was not an independent predictor of overall survival in the Cox regression; observed effects were explained by the MELD score. Overall, this suggests that IL-38 in this cohort primarily reflects the current severity without having any independent prognostic significance for long-term survival (Supplementary Figure S1A,B).

## 4. Discussion

In this prospective cohort, IL-38 emerged as a biomarker associated with the severity of liver disease. Although IL-38 concentrations differed only modestly among etiological subgroups, patients with hepatic decompensation were significantly overrepresented within the higher concentration range. In line with this observation, IL-38 alone demonstrated the ability to detect decompensation with solid diagnostic accuracy (AUC ~0.7). In parallel, IL-38 levels increased continuously with rising MELD scores and showed close associations with laboratory parameters indicative of advanced liver disease, including higher bilirubin concentrations, elevated transaminase levels, reduced platelet counts, and impaired coagulation parameters, thereby underscoring its role as a marker of the current disease burden.

It should be particularly emphasized that the combination of IL-38 with the MELD score achieved very good discriminatory performance for the presence of decompensation in a simple regression model (AUC ~0.9), thereby meaningfully complementing the established risk score concept. At the same time, our data indicate that IL-38 in this cohort functions less as an independent long-term prognostic factor and more as a reflection of current disease severity and inflammatory activation. Overall, IL-38 therefore appears promising for the assessment of decompensated disease courses and as a component of multimodal severity scores or progression parameters, and should be further validated in larger prospective cohorts.

While the disease-related anti-inflammatory and anti-fibrotic role of IL-38 has already been demonstrated in several organ systems and disease entities, the data available for liver diseases remain sparse [9,24,25,26]. In view of the close association between cardiovascular diseases and steatotic liver diseases in the context of the metabolic syndrome, the metabolically beneficial properties of IL-38 are of particular interest [27]. In murine models, IL-38 has been shown to reduce hepatic fat content and to improve glucose tolerance and insulin sensitivity [28]. In a human MASLD cohort, significantly higher IL-38 concentrations were observed in patients with MASLD compared with healthy individuals, together with positive correlations with sonographically verified hepatic steatosis, markers of insulin resistance, liver enzymes, and triglyceride levels. Given its favorable discriminatory performance, IL-38 may therefore represent a non-invasive biomarker for both the presence and severity of MASLD [15,16,28].

A study investigating IL-38 in patients with chronic hepatitis B in comparison with a healthy cohort and before and after successful antiviral therapy reported lower IL-38 concentrations in healthy controls and a normalization of these levels following treatment. Furthermore, an improved therapeutic response was observed in patients with higher baseline IL-38 concentrations, and IL-38 has therefore been discussed as a potential biomarker of treatment response [17].

In marked contrast, we observed reduced IL-38 concentrations in our SLD cohort with chronic advanced liver disease compared with healthy subjects. However, the difference in IL-38 levels between ALD/metALD patients and healthy controls reached only marginal statistical significance (*p* = 0.046), likely due to the small size of the control group, and should therefore be interpreted with caution. This observation requires confirmation in larger cohorts. Evidently, IL-38 levels do not uniformly follow the same pattern depending on disease activity and may either be reduced in disease, potentially reflecting consumption or contributing to disease progression or chronicity, or may be compensatorily elevated [17]. This interpretation is further supported by our finding of significantly higher IL-38 concentrations in decompensated patients. This may also explain the observed correlations with laboratory markers of decompensation, such as bilirubin, and the absence of an association with markers of chronic inflammation, such as C-reactive protein. Available data to date suggest that IL-38 tends to increase with the severity of the respective disease, a pattern that appears consistent across different organ systems [10]. Studies investigating IL-38 in other diseases and organ systems have similarly demonstrated that IL-38 is particularly elevated in acute disease settings, including myocardial infarction, newly diagnosed multiple sclerosis, and acute respiratory distress syndrome, and that concentrations decline in chronic conditions or following successful therapy [29,30,31]. Comparable findings have been reported for other anti-inflammatory members of the interleukin-1 family, such as interleukin-37 [20].

The association between IL-38 and advanced SLD severity observed in this study is biologically plausible in light of its function as a counter-regulatory cytokine within the IL-1 family. IL-38 exerts predominantly anti-inflammatory effects and may suppress IL-1–mediated signaling and downstream pro-inflammatory pathways [32], which is consistent with the concept that systemic inflammation represents a central driver of hepatic decompensation and adverse clinical outcomes [33,34]. Elevated IL-38 levels in advanced disease are therefore likely to reflect a compensatory response to heightened IL-1 activity rather than a direct pathogenic mechanism [32,33]. Differences in disease etiology (MASLD versus ALD), metabolic comorbidities, and the degree of hepatic dysfunction may consequently shape inflammatory profiles and circulating IL-38 concentrations [32,35,36]. Although IL-38 may act as an etiology-independent marker of decompensation across SLD, our data suggest that it primarily reflects disease severity rather than independently predicting outcome, in contrast to IL-6 [37]. In comparison, macrophage activation markers, including sCD163, sMR, and sCD206, show strong correlations with disease severity and mortality and frequently outperform established prognostic scores [38,39]. Future investigations should position IL-38 within the broader IL-1 signaling axis using longitudinal and mechanistic approaches to further clarify its counter-regulatory role in inflammation-driven hepatic decompensation [32,34,37].

This prospective single-center study is subject to potential selection bias, residual confounding, and limited generalizability. Small sample sizes within individual clinical and etiological subgroups reduced statistical power for group, interaction, and survival analyses, increased uncertainty around effect estimates, and heightened sensitivity to outliers. Consequently, relevant differences may have remained undetected, and borderline significant findings—particularly the etiology-dependent association between IL-38 and AST [40]—should be interpreted with caution and validated in larger, adequately powered cohorts. An additional limitation is the very small number of healthy control subjects, which precludes the establishment of a robust reference range for IL-38 and limits patient–control comparisons. Finally, IL-38 was assessed at a single time point, precluding evaluation of its temporal dynamics.

Because IL-38 correlates strongly with global disease severity as reflected by the MELD score, adjusted models may be affected by over-adjustment, particularly in view of collinearity with individual MELD components such as bilirubin and coagulation parameters. The performance of multiple exploratory analyses further increases the risk of type I error. Our findings, therefore, warrant confirmation in prospective, multicenter cohorts with standardized sampling procedures, repeated IL-38 measurements, comprehensive covariate assessment, and external validation of predictive models.

In addition to MASLD, our results suggest that IL-38 is associated with disease severity in ALD and metALD and may represent a candidate biomarker in these conditions. IL-38 could contribute to risk stratification and complement established prognostic scores as an additive parameter; however, its greatest value appears to reside within a multimodal framework integrating clinical and laboratory data. External validation in an independent, prospective multicenter cohort is required to confirm its clinical relevance and generalizability.

## 5. Conclusions

In our cohort, IL-38 emerged primarily as a marker of disease severity: concentrations were elevated in patients with decompensated cirrhosis and demonstrated a robust correlation with the MELD score, as well as with multiple laboratory parameters indicative of advanced liver disease. These observations suggest that IL-38 is a biologically plausible biomarker closely reflecting current disease activity and systemic inflammatory burden. Importantly, while IL-38 correlates with established markers of severity, its incremental clinical utility over existing prognostic indices has yet to be determined. Future investigations in larger, prospective, and methodologically standardized cohorts are therefore warranted to assess whether IL-38 can provide complementary information for risk stratification, guide clinical decision-making, or serve as a component of multimodal severity or progression scores in steatotic and alcohol-associated liver disease.

## Figures and Tables

**Table 1:** IL-38 pairwise comparisons vs. healthy controls.

Comparison	n1	n2	U	Z	*p*	r
ALD vs. Healthy	59	8	2608	−0.743	0.457	0.091
metALD vs. Healthy	39	8	3056	−1.873	0.061	0.273
ALD/metALD vs. Healthy	98	8	225	−1.997	0.046	0.194

Two-sided Mann–Whitney U tests; n1 and n2: valid counts per group, *p*: unadjusted *p*-values, r: effect size.

**Table 2:** Correlations between IL-38 and routine laboratory parameters.

Parameter	ρ	95% CI
PT	−0.271 **	[−0.411, −0.119]
aPTT	0.248	[−0.019, 0.482]
Alb	−0.135	[−0.300, 0.037]
GGT	0.004	[−0.153, 0.161]
ALT	0.034	[−0.123, 0.190]
AST	0.157 *	[0.000, 0.306]
Na	0.062	[−0.097, 0.218]
TBil	0.273 **	[0.122, 0.412]
ALP	0.063	[−0.096, 0.218]
Cr	0.107	[−0.050, 0.259]
Plt	−0.305 **	[−0.441, −0.155]
WBC	−0.170 *	[−0.319, −0.013]
CRP	0.095	[−0.069, 0.254]
LDH	0.179 *	[0.020, 0.329]

Spearman correlation (ρ) between ln(IL-38) and laboratory parameters. 95% confidence intervals (CI) via Fisher’s z approximation for Spearman’s ρ. Significance stars denote approximate two-sided *p*-values: ** *p* < 0.01, * *p* < 0.05. IL-38, Interleukin-38; ln, natural logarithm; PT, prothrombin time; aPTT, activated partial thromboplastin time; Alb, albumin; GGT, gamma-glutamyl transferase; ALT, alanine aminotransferase; AST, aspartate aminotransferase; Na, sodium; TBil, total bilirubin; ALP, alkaline phosphatase; Cr, creatinine; Plt, platelets; WBC, white blood cells; CRP, C-reactive protein; LDH, lactate dehydrogenase.

**Table 3:** Logistic regression for decompensation.

Model	Predictors	OR (95% CI)	*p*-Value	AUC (95% CI)
logIL-38 only	logIL-38	2.34 (1.57–3.50)	<0.001	0.692 (0.615–0.769)
MELD + logIL-38	MELD; logIL-38	MELD 1.92 (1.57–2.34); logIL-38 1.39 (0.83–2.32)	<0.001 0.207	0.920 (0.882–0.959)
MELD + logIL-38 + lnCRP	MELD; logIL-38; lnCRP	MELD 1.90 (1.56–2.33); logIL-38 1.38 (0.82–2.32); lnCRP 1.07 (0.97–1.17)	<0.001 0.227 0.178	0.925 (0.887–0.962)

Odds ratios (OR) with 95% confidence intervals reported per 1-unit increase. Only MELD remains significant after adjustment. AUC values are based on the ROC of each models predicted.

**Table 4:** ROC summary, cut-offs, and predictive values.

Predictor	AUC (95% CI)	Cut-Off	Sensitivity	Specificity	PPV	NPV
IL-38	0.713 (0.525–0.901)	0.138	0.663	0.750	0.699	0.717
Pred. prob. (logIL-38 only)	0.692 (0.615–0.769)	0.346	0.955	0.594	0.674	0.938
Pred. prob. (MELD + logIL-38)	0.920 (0.882–0.959)	0.463	0.882	0.854	0.841	0.892
Pred. prob. (MELD + logIL-38 + lnCRP)	0.925 (0.887–0.962)	0.435	0.882	0.843	0.831	0.891

Row 1 (“IL-38 (ng/mL)”) reports a ROC analysis using the raw IL-38 concentration as a standalone marker; the cut-off is therefore expressed in ng/mL (Youden-optimal threshold). Row 2 (“Predicted probability [logIL-38 only]”) reports a ROC analysis of the predicted probability from a logistic regression with log-transformed IL-38 as the sole predictor; the cut-off is therefore a probability. Both rows evaluate the same biomarker on different scales (raw concentration vs. model-based probability); minor differences in AUC can arise from sample composition/missingness and thresholding. Rows 3–4 present multivariable models (MELD ± ln-CRP), with ROC analyses performed on their predicted probabilities and Youden-optimal probability thresholds. PPV/NPV are derived from sensitivity, specificity, and the overall prevalence of decompensation in this cohort and will vary with prevalence.

## Data Availability

The data presented in this study are available on request from the corresponding author due to ethical and institutional restrictions.

## Supplementary Materials

The following supporting information can be downloaded at: https://www.mdpi.com/article/10.3390/cells15030280/s1, Figure S1: Overall survival in the ALD/metALD cohort according to IL-38; Table S1: Baseline characteristics by disease.

## Abbreviations

ACLD	Advanced chronic liver disease
ACLF	Acute on chronic liver failure
ALD	Alcohol-associated liver disease
Alb	Albumin
ALP	Alkaline phosphatase
ALT	Alanine aminotransferase
aPTT	Activated partial thromboplastin time
AST	Aspartate aminotransferase
AUC	Area under the curve
cACLD	Compensated advanced chronic liver disease
CI	Confidence interval
CSPH	Clinically significant portal hypertension
Cr	Creatinine
CRP	C-reactive protein
GGT	Gamma-glutamyl transferase
HE	Hepatic encephalopathy
HVPG	Hepatic venous pressure gradient
IL	Interleukin
LDH	Lactate dehydrogenase
ln	Natural logarithm
MASLD	Metabolically associated steatotic liver disease
MASH	Metabolic Dysfunction-associated Steatohepatitis
metALD	Metabolic dysfunction and alcohol associated liver disease
PAMP/DAMP	Molecular pattern/damage associated molecular pattern
Na	Sodium
NIT	Non-invasive biomarkers
OR	Odds ratio
Plt	Platelets
PT	Prothrombin time
SLD	Steatotic liver disease
sMR	Soluble mannose receptor
TBil	Total bilirubin
Tregs	Regulatory T cells
WBC	White blood cells
