# Supplementary material for: Interleukin-38: A Candidate Biomarker for Disease Severity in Advanced Steatotic Liver Disease
Source: Cells. 2026 Feb 2;15(3):280. doi: 10.3390/cells15030280 (PMC12897045; doi:10.3390/cells15030280)
Supplement: Supplementary file 1 [file cells-15-00280-s001.zip › cells-4087202-supplementary.pdf]

Interleukin-38 as a mechanistic and predictive biomarker in advanced steatotic liver disease

Supplementary Data

Valeria Wagner[a*]; Michael Mederer[a]; Barbara Enrich [a]; Veronika Cibulkova[a]; Johanna Piater[a]; Andreas Zollner[a], Rebecca Giquel-Fernandes[a], Herbert Tilg[a]; Maria Effenberger[a]

[a] Department of Internal Medicine I, Gastroenterology, Hepatology, Endocrinology & Metabolism, Medical University of Innsbruck, 6020 Innsbruck, Austria

Correspondence: Maria Effenberger, Department of Internal Medicine I, Gastroenterology, Hepatology, Endocrinology & Metabolism, Medical University Innsbruck, Christoph-Probst-Platz 1, Innrain 52 A. Email: maria.effenberger@i-med.ac.at

Figure S1: Overall survival in the ALD/metALD cohort according to IL-38

A

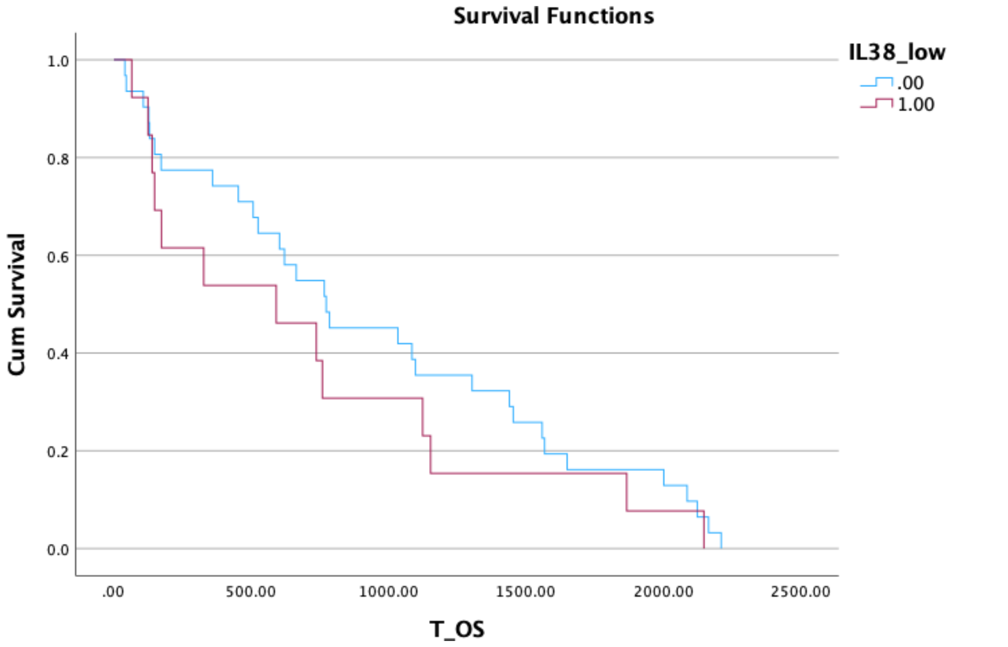

B

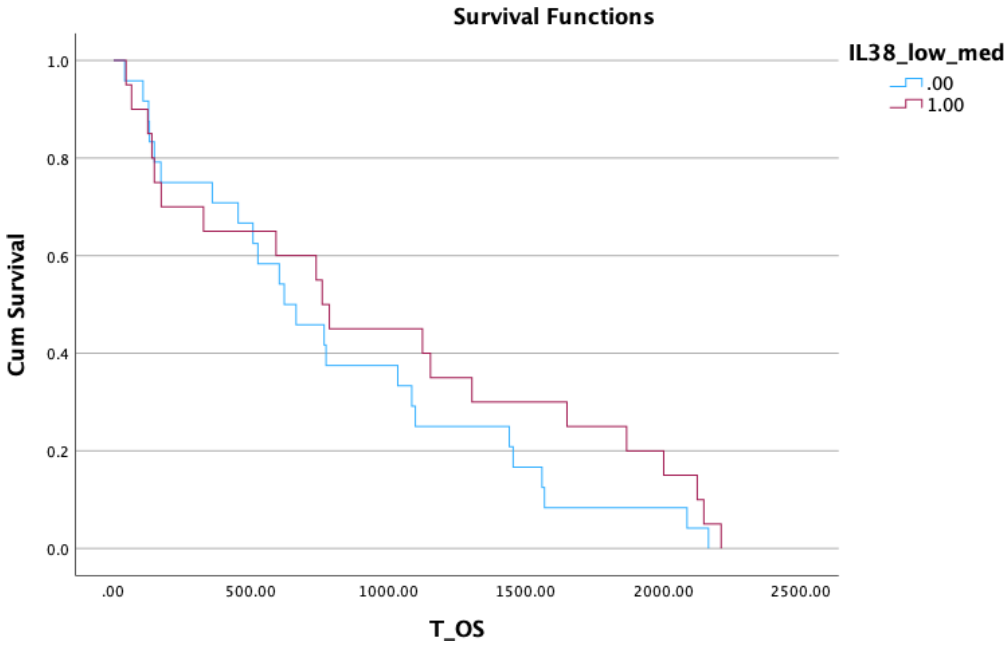

Kaplan–Meier curves for overall survival in the ALD/metALD cohort, stratified by serum IL-38 at study inclusion.

(A) Dichotomization at the cohort median (“IL-38 low” vs. “IL-38 high”).
(B) Dichotomization at the Youden cut-off from the ROC analysis for decompensation (≈ 0.138 ng/mL). The survival curves did not differ significantly (p≈0.306).

Table S1. Baseline characteristics by disease

Characteristic	Healthy	MASLD	ALD	metALD	p-value
n	8	78	59	39	
Female n (%)	4 (50.0%)	20 (25.6%)	20 (33.9%)	9 (23.1%)	0.320
BMI, kg/m² (median [IQR])	27.8 [6.2–33.8]	28.4 [24.25–31.0]	25.4 [23.0–28.5]	25.6 [22.9–30.5]	0.167
MELD (median [IQR])	n/a	9 [7–13]	12 [10–16]	11 [9–13]	0.002
Decompensated n (%)	0 (0.0%)	28 (35.9%)	39 (66.1%)	19 (48.7%)	<0.001
IL-38, ng/mL (median [IQR])	0.161 [0.092–0.299]	0.174 [0.100–0.339]	0.187 [0.150–0.294]	0.135 [0.097–0.323]	0.099
Height in cm (median [IQR])	170 [158–186]	175 [166–181]	172 [168–178]	172 [166–178]	0.580
Weight in kg (median [IQR])	78.7 [19.1–112.5]	81.1 [75.25–97.5]	76.0 [67.0–84.0]	76.4 [64.25–93.0]	0.011

Continuous variables summarized as median [IQR]; categorical as n (%). Overall p-values: Kruskal–Wallis for continuous variables, Pearson’s correlation for categorical variables. MELD not applicable in healthy controls.
